# Influence of long-term storage temperatures and sodium fluoride preservation on the stability of synthetic cathinones and dihydro-metabolites in human whole blood

**DOI:** 10.1007/s11419-022-00634-w

**Published:** 2022-08-06

**Authors:** Abdulaziz A. Aldubayyan, Erika Castrignanò, Simon Elliott, Vincenzo Abbate

**Affiliations:** 1 Department of Analytical, Environmental and Forensic Sciences, Faculty of Life Sciences and Medicine, King’s College London, London, UK; 2 Department of Toxicology, Central Military Laboratory and Blood Bank, Prince Sultan Military Medical City, Riyadh, Saudi Arabia; 3 Elliott Forensic Consulting, Birmingham, UK

**Keywords:** LC–MS/MS, Synthetic cathinones, Stability, Whole blood, New psychoactive substances, Dihydro-metabolites

## Abstract

**Purpose:**

Synthetic cathinones, one of the largest groups of new psychoactive substances, represent a large analytical and interpretative challenge in forensic laboratories. Of these is the synthetic cathinones’ instability in different biological samples, which may lead to drug concentration discrepancies when interpreting toxicological findings. In this study, the stability of a panel of synthetic cathinones and their dihydro-metabolites (*n* = 26) together with internal standard was monitored in human whole blood stored at various temperatures over 6 months. The influence of sodium fluoride as a preservative in blood collection tubes was also investigated.

**Methods:**

Samples were extracted using a two-step liquid-liquid extraction technique, and analyzed using a validated liquid chromatography–tandem mass spectrometry method following recommendations of published guidelines.

**Results:**

The influence of temperature over analytes’ stability was an important element in whole blood samples, with − 40 °C being the best storage temperature for all tested analytes. Sodium fluoride did not significantly affect the stability of cathinones except at room temperature. Dihydro-metabolites displayed better stability in whole blood samples and remained detectable for a longer period of time under all tested conditions.

**Conclusions:**

The data suggest that samples containing synthetic cathinones should be analyzed immediately, if possible. Alternatively, whole blood samples should be stored frozen (at − 40 °C or lower); however, (quantitative) results should be interpreted with caution after long-term storage. The data also promote the use of dihydro-metabolites as biomarkers for synthetic cathinones intake, as these reduced metabolites may be detected for longer period of time when compared with parent drugs in whole blood samples.

**Supplementary Information:**

The online version contains supplementary material available at 10.1007/s11419-022-00634-w.

## Introduction

Synthetic cathinones (SCt), a class of new psychoactive substances (NPS), are structurally derived from cathinone, which is the principal psychoactive component of the “Khat” plant (*Catha edulis*) [1]. These drugs, also known as “legal highs’ since they were initially legal in Europe and the UK, are often sold under misleading names like “bath salts”, “research chemicals”, or “plant food” [2, 3]. Owing to the constant/dynamic evolution of new uncontrolled derivatives to replace those controlled by the law, these drugs became an attractive alternative to classical stimulant drug users [4]. Currently, SCt are one of the largest groups of NPS being monitored by the United Nation Office of Drugs and Crime (UNODC) [5].

Whole blood is one of the preferred samples in most forensic laboratories owing to its ability to reflect the concentration of a drug/poison at the time of death or within a short time prior to sample collection in life. Recently, Adamowicz [6] reviewed the reported SCt concentrations’ in whole blood in both nonfatal intoxications and postmortem cases. Among the reviewed cases, 2-methylmethcathinone (2-MMC) concentration range in nonfatal cases was 1–13 ng/mL; 3,4-methylenedioxypyrovalerone (MDPV), 1–8400 ng/mL; 3,4-methylenedioxy-α-pyrrolidinobutyrophenone (MDPBP), 2–92 ng/mL; 3-methylethcathinone (3-MEC), 32–332 ng/mL; 4-chloroethcathinone (4-CEC), 2 ng/mL; 4-fluoro-α-pyrrolidinohexanophenone (4-F-PHP), < 1–28 ng/mL; 4-methylethcathinone (4-MEC), 28–353 ng/mL; butylone, 250–910 ng/mL, whereas concentration of mephedrone in postmortem cases ranged from 2 to 22,000 ng/mL; 3,4-dimethylmethcathinone (3,4-DMMC), 53 to 27,000 ng/mL; MDPV, < 1–29,000 ng/mL; 4-chloro-α-pyrrolidinovalerophenone (4-Cl-α-PVP), 9 to 11 ng/mL; 4-F-PHP, 13 ng/mL.

However, drug stability is a concern in blood when compared to other biological specimens owing to the expected enzymatic reactions and activity following sampling [7]. Indeed, a few days to weeks can pass between sampling and analysis mainly due to delay in transportation, other logistical considerations, and laboratory workload. Moreover, forensic laboratories have to store evidence after toxicological analyses are performed to enable reanalysis if requested [7]. Instability of analytes can be problematic in such situations, and therefore, knowledge of the analytes’ stability in biological samples is of great value in forensic cases, since it could aid better interpretation of the results.

Various studies have been performed to investigate the stability of SCt in whole blood. Glicksberg and Kerrigan [8] studied the stability of 22 cathinone derivatives in preserved whole blood (1% sodium fluoride as preservative) at 32, 20, 4, and − 20 °C for 6 months. They concluded that analytes belonging to the group of tertiary amines with MD substitution (methylenedioxy-substituted) (e.g., MDPV, 3,4-methylenedioxy-α-pyrrolidinobutiophenone) were shown to be the most stable at all tested storage conditions, with − 20 °C being the optimal storage temperature. Busardò et al*.* [9]*,* evaluated the importance of stabilizers in human whole blood. Mephedrone was stored at − 20, 4, and 20 °C for 185 days with different stabilizers such as sodium fluoride/potassium oxalate (NaF/KOx), ethylenediaminetetracetate (EDTA), and without additives. Mephedrone was stable at − 20 °C, but greater stability was noted with NaF/KOx followed by EDTA, when compared to samples stored without additives. In addition to the parent mephedrone, Czerwinska et al*.* [10] reported the stability of five phase I metabolites with dihydro-mephedrone and dihydro-normephedrone being the most stable in whole blood (containing NaF/KOx) when stored at 4 and − 20 °C for 10 days. Soh and Elliott [11] reported a completed degradation of 4-MEC in whole blood after 14 days at room temperature (RT), while dihydro-4-MEC (following reduction of ketone group) was identified to be a breakdown product in this matrix.

Longer stability data of the dihydro-metabolites and other SCt derivatives (e.g., 4-chloroethcathinone, *N*-ethylhexedrone, 4-methylpentedrone, 4-fluoro-α-pyrrolidinohexanophenone, 4-chloro-α-pyrrolidinopropiophenone and 4‐chloro‐α‐pyrrolidinovalerophenone) in whole blood have not been reported before. Additionally, data on the effect of preservative on stability of SCt in whole blood are very limited.

**Fig. 1:**
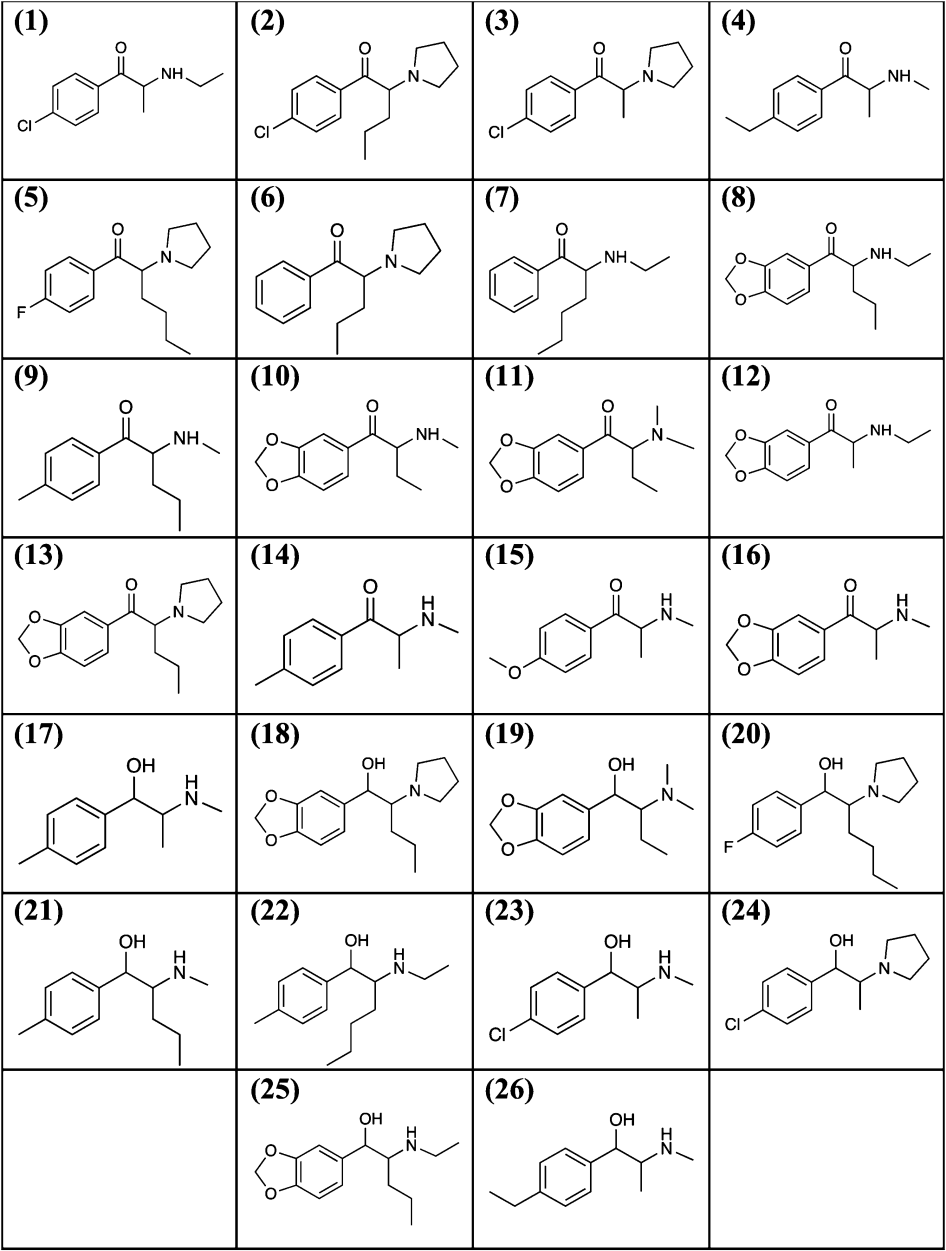
Chemical structures of **(1)** 4-CEC, **(2)** 4-Cl-α-PVP, **(3)** 4-Cl-α-PPP, **(4)** 4-EMC, **(5)** 4-F-PHP, **(6)** α-PVP, **(7)** *N*-ethylhexedrone, **(8)** *N*-ethylpentylone, **(9)** 4-MPD, **(10)** butylone, **(11)** dibutylone, **(12)** ethylone, **(13)** MDPV, **(14)** mephedrone, **(15)** methedrone, **(16)** methylone, **(17)** dihydro-mephedrone, **(18)** dihydro-MDPV, **(19)** dihydro-dibutylone, **(20)** dihydro-4-F-PHP, **(21)** dihydro-4-MPD, **(22)** dihydro-*N*-ethylhexedrone, **(23)** dihydro-4-CEC, **(24)** dihydro-4-Cl-α-PPP, **(25)** dihydro-*N*-ethylpentylone, and **(26)** dihydro-4-EMC involved in the study

This study presents (i) a validated liquid chromatography–tandem mass spectrometry (LC–MS/MS) method for the detection and quantification of a total of 26 synthetic cathinones and metabolites in human whole blood (Fig. 1) and (ii) its application to a 6 month long-term stability study of these analytes in whole blood. The stability assessed the influence of storage temperatures (room temperature, 4, − 20, and − 40 °C) and preservative (with and without NaF) under the typical conditions encountered in forensic laboratories.

## Materials and methods

### Chemicals and reagents

Reference standards of 4-CEC, 4-EMC, butylone, dibutylone, ethylone, MDPV, mephedrone, methedrone, methylone, *N*-ethylhexedrone, *N*-ethylpentylone, and α-PVP were purchased from Chiron (Surrey, UK). 4-MPD, 4-F-PHP, 4-Cl-α-PPP, and 4-Cl-α-PVP were kindly donated by TicTaC communications (London, UK) and their structures were confirmed via high-resolution mass spectrometry (HRMS). Internal standard (IS) solution of MDPV-*d*_8_ (0.1 mg/mL) was purchased from Sigma-Aldrich (Dorset, UK). All reference standards were obtained as methanolic solutions at 1.0 mg/mL, except for mephedrone, 4-MPD, 4-F-PHP, 4-Cl-α-PPP, and 4-Cl-α-PVP (1 mg of powder). Drug-free human whole blood (in 10 mL tubes) (containing K_2_EDTA) was purchased from Cambridge Bioscience (Cambridge, UK).

Water was purified on a Milli-Q system (Burlington, MA, USA). All solvents were analytical or high-performance liquid chromatography (HPLC) grade. Methanol (MeOH) and acetonitrile (ACN) were obtained from Sigma-Aldrich (Dorset, UK). 1-Chlorobutane and sulphuric acid (≥ 95%) both were obtained from Thermo Fisher Scientific (Loughborough, UK).

### Preparation of standards and solutions

Mixed analyte working solutions were prepared by dilution of reference standards in MeOH to establish calibrators at 2, 10, 20, 40, 100, 1000, 1500, and 2000 ng/mL. Likewise, working solutions for the quality controls (QCs) were diluted in MeOH at 60, 800, and 1600 ng/mL. The IS solution (MDPV-*d*_8_) was diluted in MeOH to achieve 2000 ng/mL. All solutions were stored at − 20 °C.

Matrix-matched calibration standards consisting of 1, 5, 10, 20, 50, 150, 500, 750, and 1000 ng/mL were prepared by the addition of an appropriate volume of the standard working solution to blood. Similarly, QCs at multiple concentrations levels, low (30 ng/mL), medium (400 ng/mL), and high (800 ng/mL), were prepared by the addition of an appropriate volume of the QC working solution to blood. An appropriate volume of IS at 2000 ng/mL was added in sodium carbonate solution to achieve the final concentration of 500 ng/mL.

### Synthesis of reduced metabolites

SCt are metabolized to several phase I metabolites, one of which is their keto-reduced metabolite (dihydro-), which may also be an instability product. However, many such metabolites are not commercially available as reference standards. Dihydro-metabolites were therefore synthesized from corresponding parent drugs, in which the keto (C=O) group was reduced to an alcohol moiety (C–OH) following a previously described method [12]. As a proof of this approach, the reduction of mephedrone to dihydro-mephedrone was studied as a starting analyte and the results were compared with the previously published data [13]. Briefly, the experimental synthetic procedure was as follows: 8 mg of sodium borohydride was added carefully and in small portions to a solution of mephedrone (0.4 mg, 2.25 × 10^–6^ mmol) in MeOH (4 mL). The solution was left overnight with agitation at room temperature. Then, the resultant mixture was dried under vacuum, and the solid residue was partitioned in dichloromethane/water (4 mL) and the organic layer was extracted (2 × 3 mL of water). Thereafter, 20 mg of sodium sulfate were added to the combined and isolated organic layer; the solution was filtered and dried under vacuum leading to dihydro-mephedrone (0.404 mg, 2.25 × 10^–6^ mmol calculated as a theoretical 100% yield based on literature findings and due to inability to accurately measure the amount of product). The solid residue was dissolved in 4.04 mL MeOH to achieve an estimated 100 μg/mL stock solution. The product ion spectra were in accordance with reported literature, and therefore, the method was applied to the following selected analytes: dibutylone, 4-CEC, 4-Cl-α-PPP, 4-EMC, *N*-ethylpentylone, MDPV, 4-MPD, *N*-ethylhexedrone, and 4-F-PHP.

### Extraction method

Liquid-liquid extraction (LLE) was performed by the addition of 0.5 mL of whole blood containing standard/QC into 15 mL polypropylene falcon tubes and fortified with 0.5 mL of 0.2 M sodium carbonate (pH = 10) containing IS. Drug-free (blank) blood was used for a ‘zero’ blank sample.

Five milliliters of 1-chlorobutane was then added to all samples, vortexed for 3 min, and then centrifuged for 5 min at 3000 rpm. The supernatant was added to new 15 mL polypropylene tubes and back extraction solvent (0.1 mL of 0.05 M sulphuric acid) was added. Samples were re-mixed for 3 min, followed by centrifugation for 5 min at 3000 rpm. The supernatant was decanted by freezing the aqueous layer and 0.1 mL of the final extract was transferred into an HPLC vial for injection.

### Instrumental analysis

Analysis of samples was performed on an Acquity UPLC^®^ system (Waters, Manchester, UK) coupled to a Waters Quattro Premier XE™ triple quadrupole (QqQ) mass spectrometer system (Waters) in positive electrospray ionization mode. The separation was performed on HSS T3 UPLC analytical column (150 × 2.1 mm, 100 Å, 1.8 μm) kept at a temperature at 20 °C. A binary gradient system was used to separate analytes with mobile phase consisted of ultra-pure water (A) and ACN (B), both containing formic acid (0.1% v/v). The gradient elution started at 90% A (0–1.80 min), decreased to 64% A (1.80–6.0 min), and then further decreased to 0% A (6.0–9.80 min). This was hold for 1 min (9.80–10.80 min). Within 0.1 min A was returned to starting condition, i.e., 90% (10.80–10.81 min), and maintained until the end of the run (10.81–13.00 min) to re-equilibrate the column. The flow rate was set at 0.3 mL/min and the injection volume was 10 μL.

The electrospray voltage of the mass spectrometer was set at 1.5 kV, desolvation temperature at 400 °C, and the source temperature at 120 °C. Nitrogen was used as the desolvation and the cone gas, which were set at 750 and 50 L/h, respectively. Argon was used as the collision gas, at a flow rate of 0.2 mL/min, which typically gave pressures of 2.14 × 10^–3^ mbar. Tandem mass spectrometry data were collected in multiple reaction monitoring (MRM) mode with two transition ions per parent analyte (one as quantifier and one as qualifier) and one MRM for each reduced metabolite (as quantifier) and IS (Table 1). The dwell time was optimized for each transition ion to obtain at least 12 data points per chromatographic peak. Data were acquired with MassLynx 4.1 software and processed with QuanLynx 4.1 software (Waters).

**Table 1:** Multiple reaction monitoring conditions for analysis of 26 synthetic cathinones, dihydro-metabolites and  internal standard (MDPV-*d*_8_) in whole blood by liquid chromatography–tandem mass spectrometry

Analyte number	Target analyte	Rt (min)	Precursor ion (*m/z)*	Product ion(s) (*m/z)*	CV (V)	CE (eV)	Dwell time (s)
1	Methylone	5.43	208	**160**	22	17	0.150
				132		25	
2	Ethylone	5.82	222	**174**	24	19	0.050
				146		24	
3	Methedrone	5.82	194	**161**	23	19	0.010
				176		12	
4	Butylone	6.12	222	**146**	24	24	0.020
				204		13	
5	Dihydro-mephedrone	6.08	180	**131**	24	18	0.050
				–		–	
6	Dihydro-dibutylone	6.20	238	**220**	26	12	0.050
				–		–	
7	Dibutylone	6.24	236	**86**	26	21	0.050
				191		15	
8	Mephedrone	6.23	178	**145**	24	20	0.050
				160		12	
9	4-CEC	6.78	212	**159**	27	18	0.050
				194		13	
10	Dihydro-4-CEC	6.85	214	**181**	23	23	0.050
11	Dihydro-*N*-ethylpentylone	6.93	252	**191**	27	23	0.010
				**–**		–	
12	Dihydro-4-EMC	6.96	194	**117**	26	22	0.010
13	4-Cl-α-PPP	7.01	238	**139**	30	27	0.010
				98		25	
14	4-EMC	7.05	192	**145**	26	21	0.010
				174		12	
15	*N*-ethylpentylone	7.05	250	**202**	27	18	0.010
				232		13	
16	Dihydro-4-Cl-α-PPP	7.09	240	**207**	30	22	0.010
				–		–	
17	α-PVP	7.14	232	**91**	35	25	0.010
				105		25	
18	MDPV-*d*_8_	7.24	284	**134**	33	26	0.020
				–		–	
19	MDPV	7.25	276	**126**	30	27	0.010
				135		24	
20	Dihydro-4-MPD	7.29	208	**147**	25	22	0.010
				–		–	
21	Dihydro-MDPV	7.32	278	**217**	30	22	0.010
				–		–	
22	4-MPD	7.36	206	**188**	25	13	0.010
				145		20	
23	*N*-Ethylhexedrone	7.57	220	**202**	27	14	0.010
				91		22	
24	Dihydro-*N*-ethylhexedrone	7.65	222	**147**	27	23	0.010
				–		–	
25	4-F-PHP	7.99	264	**109**	35	25	0.050
				140		30	
26	4-Cl-α-PVP	8.01	266	**125**	31	21	0.050
				139		24	
27	Dihydro-4-F-PHP	8.09	266	**109**	35	25	0.050
				–		–	

Quantifier ions in bold

*Rt* retention time, *CV* cone voltage, *CE* collision energy

### Method validation

The method was validated following the recommendations for quantitative analysis in the Scientific Working Group for Forensic Toxicology (SWGTOX) guideline that recently evolved into ANSI/ASB standard 036 [14, 15].

#### Linearity

Linearity was evaluated over five independent runs, with eight calibration points (1, 5, 10, 20, 50, 150, 500, and 1000 ng/mL for parent drugs; 1, 5, 10, 20, 50, 150, 750, and 1000 ng/mL for metabolites), except for methedrone, butylone, 4-Cl-α-PPP, *N*-ethylpentylone, 4-EMC, and MDPV that had seven calibrators (5, 10, 20, 50, 150, 750, and 1000 ng/mL). Each calibrator was required to be within ± 20% of target concentration with coefficient of determination (*R*^2^) required to be ≥ 0.990 for each target analyte to meet acceptance criteria. A linear regression model with a 1/*x* weighting was applied to the calibration curve.

#### Carryover

Blank whole blood were extracted in triplicate and analyzed after the highest calibrator (1000 ng/mL) to assess the carryover. If no peak of target analyte exceeded its limit of detection (LOD), carryover was deemed to be absent.

#### Bias and precision

Bias was assessed at three concentrations: QC low (30 ng/mL), QC medium (400 ng/mL), and QC high (800 ng/mL), with three replicates over five independent runs along with the batch of calibration curve.

Intraday and interday precisions were assessed as the percent coefficient variation (%CV) of findings from replicate samples (i.e., QC low, QC medium, and QC high) within a day (*n* = 3) and over 5 days (*n* = 15). One-way analysis of variance (ANOVA) was used to carry out calculation of precisions. Bias and precisions within ± 20% at each concentration were considered acceptable.

#### LOD and LOQ

The LOD was the lowest concentration at which analytes could produce a signal-to-noise (*S*/*N*) ratio of at least 3. Limit of quantification (LOQ) was defined as the lowest concentration of analyte that could reliably produce quantitative results with S/N ratio of at least 10. The lowest concentration of calibration curve (1 or 5 ng/mL; depending on analyte) was assigned as the LOQ of the method.

#### Interference

Interference from endogenous components was assessed using extracted blank whole blood in the absence of analytes and IS. To assess potential interference with the signal of analytes of interest produced from the use of IS, blank blood was fortified with IS (500 ng/mL) and MRM peaks of the target analytes were monitored. Equally, potential interference with the signal of IS from the analytes of interest was evaluated by analyzing highest calibrator (1000 ng/mL) in the absence of IS. Potential exogenous interference was evaluated by fortifying two control solutions (A and B) containing frequently encountered drugs (*n* = 196) in blank blood at 10 or 100 ng/mL. It is worth to note that control B solution contained also mephedrone; therefore, mephedrone was not considered for its selectivity (supplementary material, Table S1).

#### Matrix effects

Matrix effects (ME) were assessed by averaging the peak areas of five blank whole blood fortified with all target analytes at low and high QC after the extraction and compared to the average peak areas (*n* = 5) of all analytes in neat methanolic solution.

ME values of < 100% was indicative of ion suppression whereas values of > 100% was indicative of ion enhancement. ME within 25% ion suppression or enhancement and %CV within 15% was considered acceptable.

#### Processed sample stability

The stability of processed samples was assessed by extracting samples at low and high QC (*n* = 3). Samples were immediately analyzed to establish day zero concentration and left in the autosampler at 10 °C for re-analyses after 24, 48, and 72 h. The average peak area of analytes was compared with those at day zero and considered stable if results remained within ± 20%.

#### Stability study design

Unpreserved human whole blood (*n* = 8) containing K_2_EDTA were pooled together to obtain a total amount of 80 mL. The pH was measured following pooling and was 7.41. Aliquots of pooled blood were analyzed to ensure the absence of interfering substances to the signal of analytes of interest. The method showed that there was no interference from the pooled whole blood sample. Pooled blood was then fortified with all SCt and metabolites (*n* = 26) and mixed to yield a concentration of 500 ng/mL. NaF (800 mg) was added to 40 mL of fortified samples to make a 2% w/v solution and left on a rotary mixer for about 30 min to ensure the diffusion of preservative throughout the sample. All blood samples were aliquoted into Vacutainer tubes (~ 10 mL on each tube).

After aliquoting, 0.5 mL from each experimental condition were prepared in duplicate to determine baseline (T_0_) concentration with freshly prepared standards and QCs. Unpreserved (0% NaF) and preserved (2% NaF) whole blood samples were stored at four different temperatures: RT (approximate range: 20–23 °C), 4, -20, and -40 °C. At defined time points, 0.5 mL from each storage temperature and condition (i.e., unpreserved and preserved) were collected in duplicate and prepared for analysis, as shown in Table 2.

**Table 2:** Storage conditions of fortified whole blood samples for stability study (*n* = 2)

Storage temperature	Preservative condition	Analysis time points (days)
RT	Unpreserved	0, 1, 2, 3, 7, 30, 90, 180
RT	2% NaF	0, 1, 2, 3, 7, 30, 90, 180
4 °C	Unpreserved	1, 2, 3, 7, 30, 90, 180
4 °C	2% NaF	1, 2, 3, 7, 30, 90, 180
− 20 °C	Unpreserved	1, 2, 3, 7, 30, 90, 180
− 20 °C	2% NaF	1, 2, 3, 7, 30, 90, 180
− 40 °C	Unpreserved	1, 2, 3, 7, 30, 90, 180
− 40 °C	2% NaF	1, 2, 3, 7, 30, 90, 180

*RT* room temperature

#### Data analysis

The analytes’ concentration was determined based on the calibration curve created for each batch of samples. Obtained data were imported to Microsoft Excel to perform analysis. Baseline concentration (T_o_) was averaged and regarded as 100% and the percentage remaining for each time point was compared to baseline concentration. Concentrations deviating > 20% from baseline concentration at each sampling point were considered unstable. The results were statistically determined using the GraphPad Prism 8.0 (GraphPad Software, San Diego, CA, USA). Differences between unpreserved and preserved whole blood (2% NaF) and between frozen conditions (i.e., − 20 and − 40 °C) for each storage condition at each time point were assessed by applying ANOVA followed by post hoc Tukey’s test. Results with* P* < 0.05 were considered significant.

## Results

### Method validation

A linearity of all analytes was achieved in whole blood samples from 1 or 5 ng/mL to 1000 ng/mL with a 1/*x* weighted linear regression model. A set of seven or eight calibration curves yielded a coefficient of determination (*R*^2^) > 0.995 (supplementary material, Table S2).

No carryover was observed when blank samples (*n* = 3) were injected after the highest calibrator (1000 ng/mL).

**Fig. 2:**
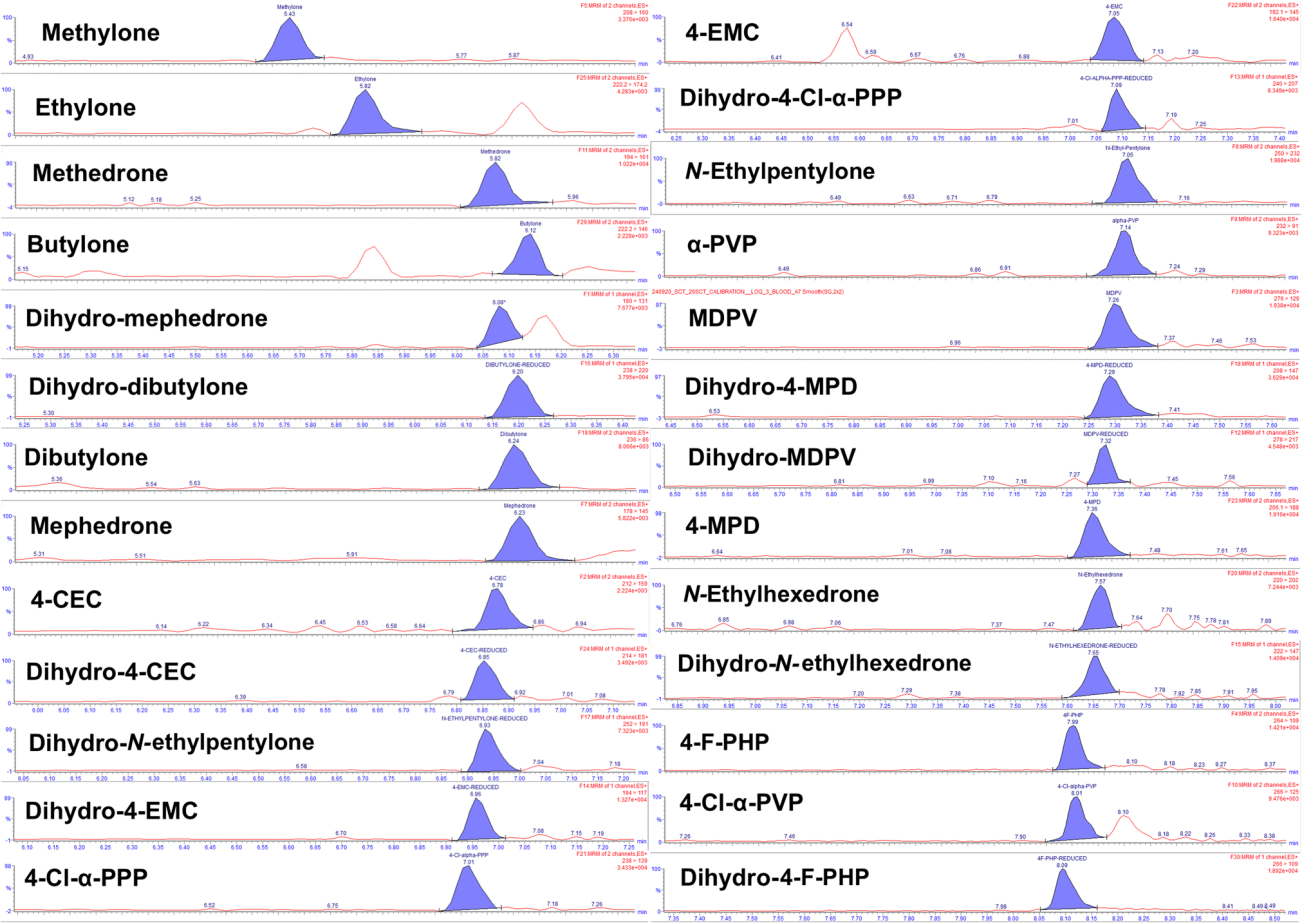
Liquid chromatography–tandem mass spectrometry chromatograms with quantifier multiple reaction monitoring transitions (see Table 1) of the analytes (*n* = 26) in whole blood at the limit of quantification (1 or 5 ng/mL)

LOD for all analytes ranged from 0.1 (4-MPD) to 1.45 ng/mL (methedrone), and LOQ was 1 ng/mL for all analytes, except for methedrone, butylone, 4-Cl-α-PPP, *N*-ethylpentylone, 4-EMC, and MDPV (5 ng/mL) (supplementary material, Table S2). Figure 2 shows MRM chromatograms of the quantifier ions for all analytes at the LOQ.

Bias ranged from -16.3 to 17.3% of the nominal concentration. Intraday %CV were < 14.2% (QC low); < 11.4% (QC medium); < 7.3% (QC high). Interday %CV for the same concentrations were < 14.9%; < 12.1%; < 7.6%. Bias and precision at the three QC concentration levels were within the acceptance limits (supplementary material, Table S3).

No endogenous interference from blank whole blood samples (*n* = 11) was observed with target analytes and IS. No interfering peak associated with the signal of target analytes for blank blood fortified with IS was observed. No IS was detected when QC high was analyzed (in the absence of IS), indicating no interfering peak associated with IS. Method selectivity was demonstrated when no signal of target analytes and IS was observed in the presence of potentially interfering drugs (control A and B), except for control B solution that contained mephedrone; therefore, mephedrone was not considered for its selectivity (supplementary material, Table S1).

Analytes had relatively low effects of ion suppression and enhancement ranging from 75.2 to 119% and from 80.9 to 108% at low and high concentration, respectively. The %CV for all analytes was < 15%, indicating no critical variations of ME from different whole blood sources (supplementary material, Fig. S1).

The stability experiments demonstrated that the processed samples were stable when kept on the autosampler at 10 °C for 24, 48, and 72 h.

### Long-term stability

#### Stability at RT

**Fig. 3:**
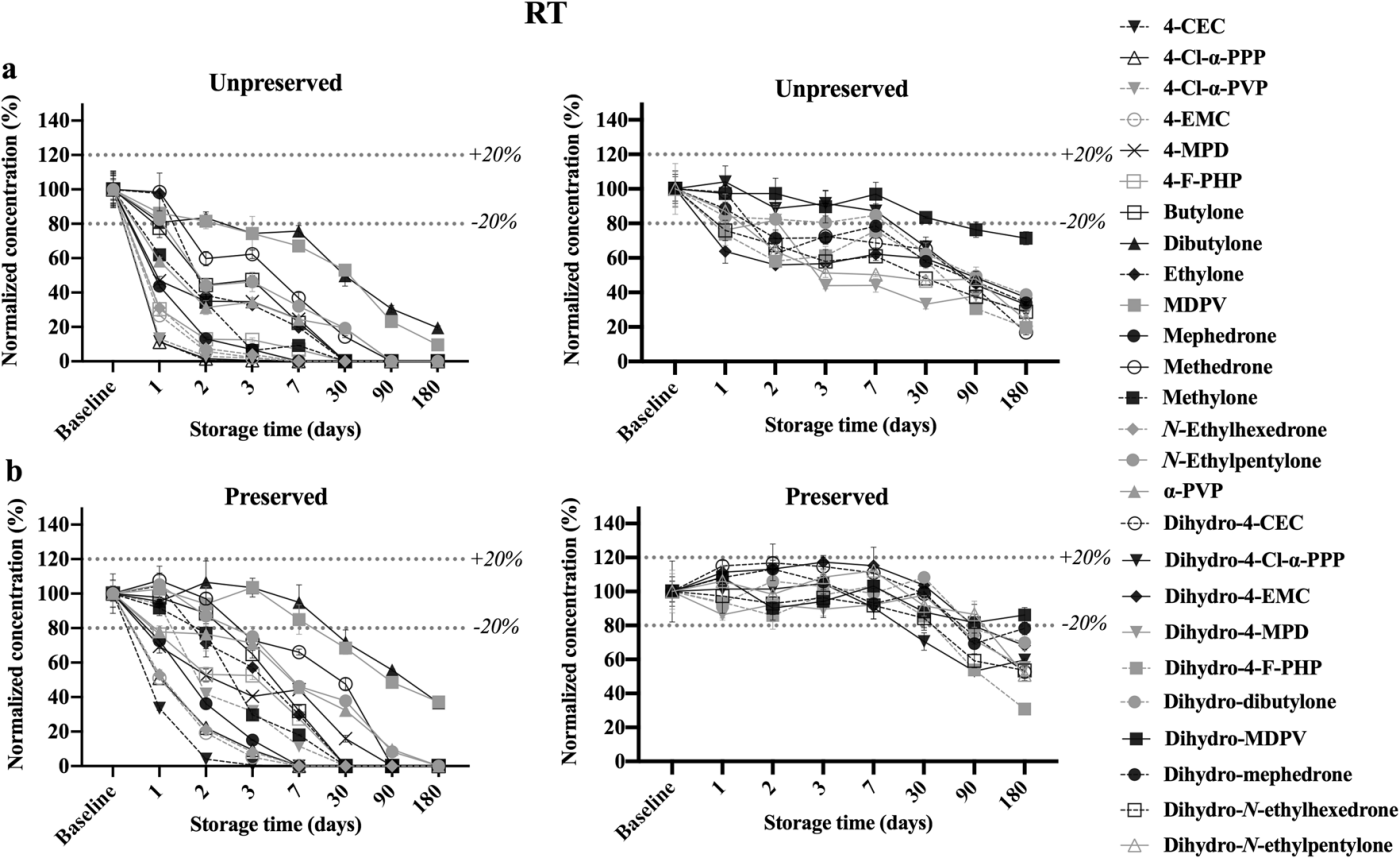
Concentrations remaining after storage at room temperature (RT) for 26 analytes and dihydro-metabolites at 500 ng/mL over 6 months. All concentrations were calculated as percentages of the baseline concentrations on T_0_. **a** Parent analytes (left) and dihydro-metabolites (right) in unpreserved whole blood. **b** Parent analytes (left) and dihydro-metabolites (right) in preserved whole blood

The stability of the parent analytes and dihydro-metabolites over the course of 6 months when stored at RT is summarized in Fig. 3. Parent SCt displayed significant instability (< 80% remaining) after 3 days of storage, where a 100% loss was observed within 90 days of storage in both unpreserved and preserved whole blood. Exceptions were dibutylone and MDPV that remained detectable over the course of 6 months (< 40% detected with NaF and < 20% detected without NaF). In contrary, 4-CEC showed the worst stability with only ~ 11 and ~ 33% (in unpreserved and preserved, respectively) of its concentration remaining after 1 day of storage, and a complete degradation after 3 days in sample without preservative; however, a measured concentration was still detectable (0.6% remaining) in sample containing NaF.

All dihydro-metabolites exhibited a degree of instability (< 80% remaining) whether with or without preservative, except for dihydro-MDPV in preserved whole blood, being stable over the 6 months. Furthermore, in contrast to the parent analytes, all metabolites had less than 40% remaining of the initial concentration in unpreserved whole blood after 6 months of storage, while preserved whole blood had more than 50% remaining.

#### Stability at 4 °C

**Fig. 4:**
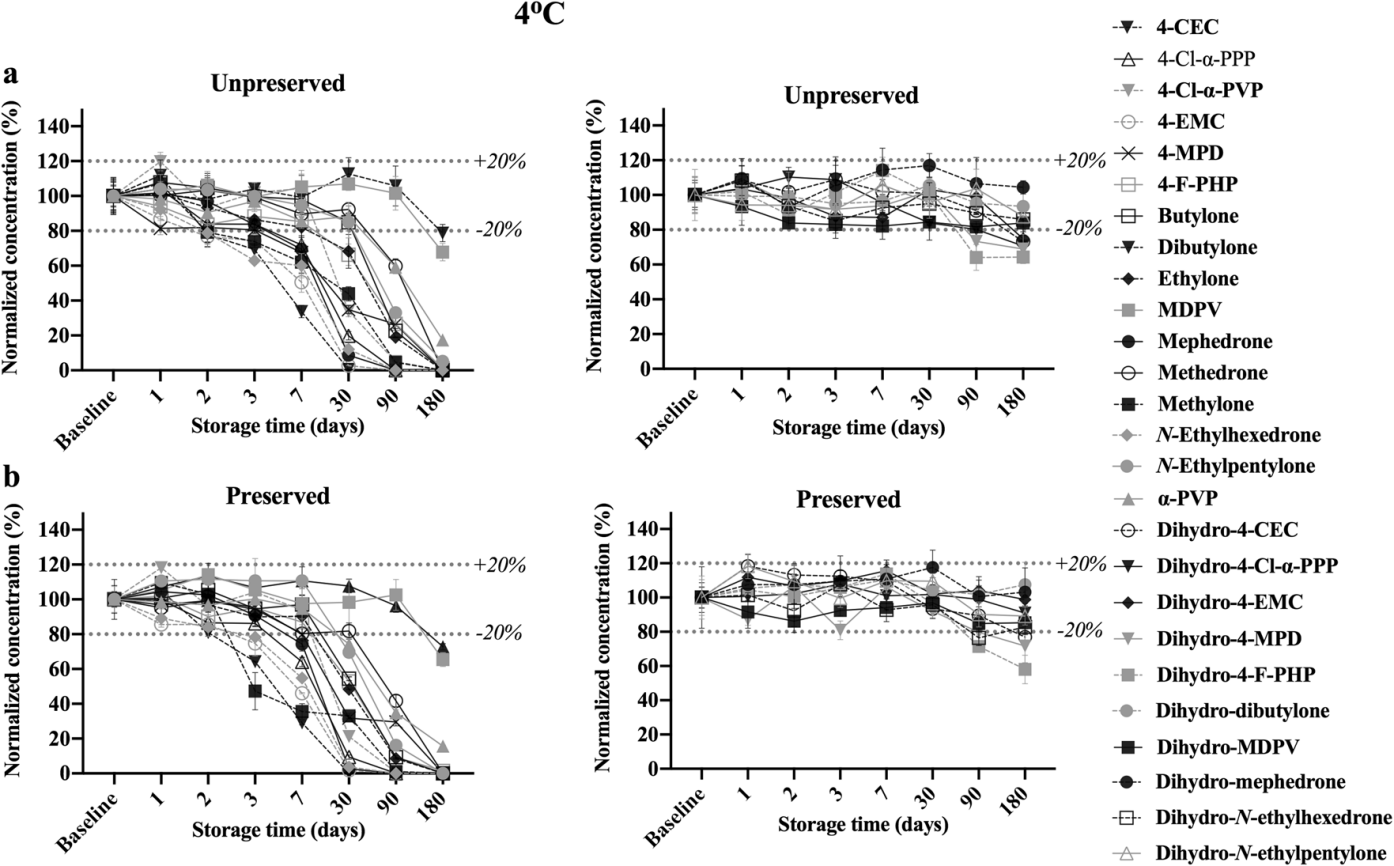
Concentrations remaining after storage at 4 °C for 26 analytes and dihydro-metabolites at 500 ng/mL over 6 months. All concentrations were calculated as percentages of the baseline concentrations on T_0_. **a** Parent analytes (left) and dihydro-metabolites (right) in unpreserved whole blood. **b** Parent analytes (left) and dihydro-metabolites (right) in preserved whole blood

The stability of parent analytes and dihydro-metabolites over the course of 6 months when stored at 4 °C is presented in Fig. 4. Most of the refrigerated analytes in whole blood samples decreased over time, but at a slower rate of degradation in this condition in comparison to RT. All analytes had greater than 60% in its content after 7 days of storage either with or without NaF. Only MDPV and dibutylone (both in the presence or absence of preservative) met the set stability criteria until day 180. However, total degradation did occur in 4-CEC, 4-EMC, mephedrone, *N*-ethylhexedrone, 4-Cl-α-PPP and in *N*-ethylpentylone, methylone, 4-F-PHP, 4-MPD, 4-Cl-α-PVP, ethylone, and butylone after 90, and 180 days, respectively, except for α-PVP that was still detectable until the end of the study (about 16% remaining for unpreserved whole blood and 17% for preserved whole blood).

Most of the metabolites were stable (> 80% remaining) under this condition throughout the 180 days despite preservation, except for 4-MPD, *N*-ethylhexedrone, and 4-F-PHP metabolites after 90 days and for 4-Cl-α-PPP, 4-CEC metabolites after 180 days. However, no complete degradation occurred after 180 days, and the remaining contents for all metabolites were greater than 60% remaining of the original concentrations in both preserved and unpreserved whole blood.

#### Stability at − 20 °C

**Fig. 5:**
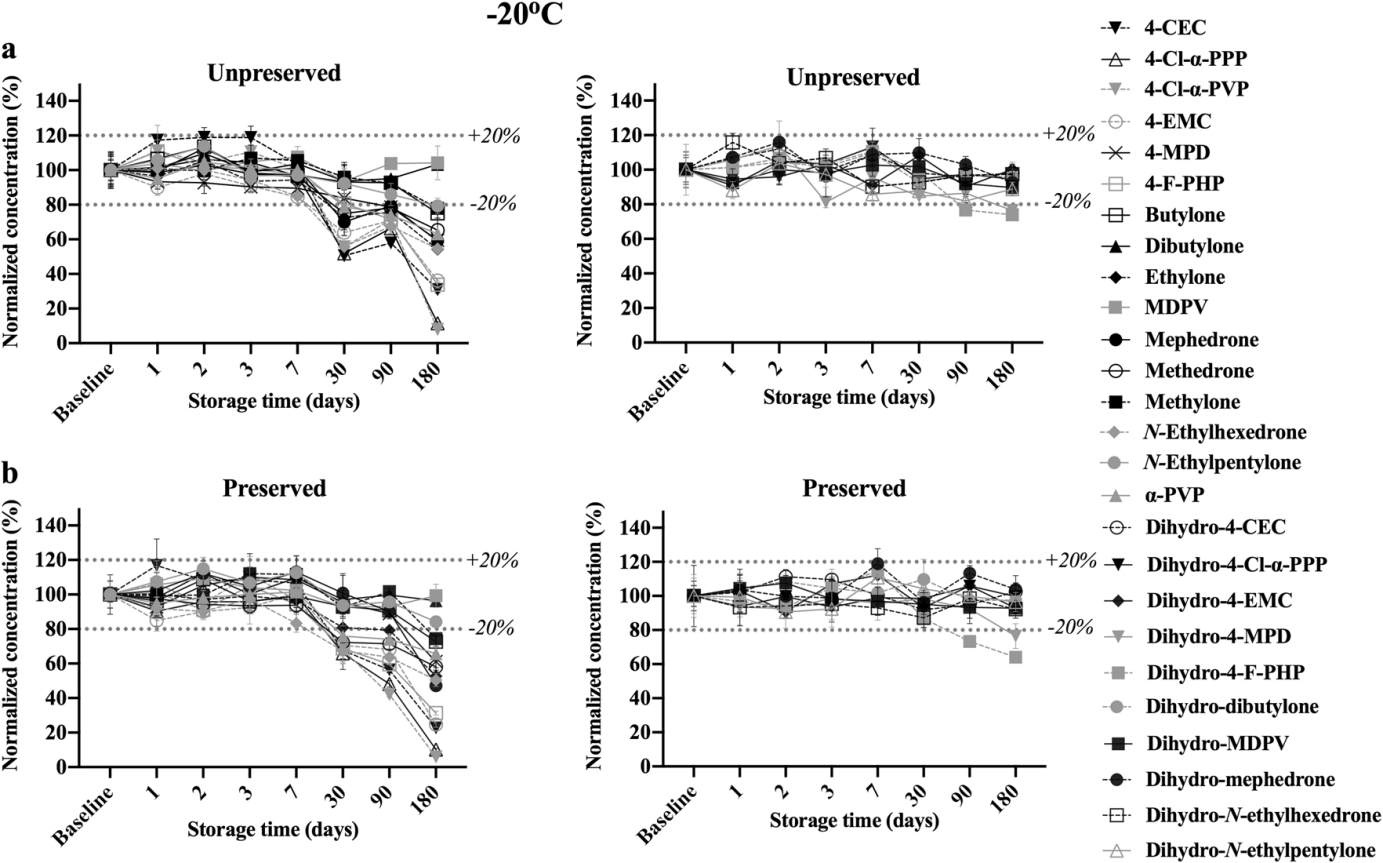
Concentrations remaining after storage at − 20 °C for 26 analytes and dihydro-metabolites at 500 ng/mL over 6 months. All concentrations were calculated as percentages of the baseline concentrations on *T*_0_. **a** Parent analytes (left) and dihydro-metabolites (right) in unpreserved whole blood. **b** Parent analytes (left) and dihydro-metabolites (right) in preserved whole blood

The stability of parent analytes and dihydro-metabolites over the course of 6 months when stored at − 20 °C is provided in Fig. 5. All analytes were found to be fairly stable for 7 days before a significant decline in concentrations was observed on day 30. Exceptions were MDPV, dibutylone, 4-MPD, and methylone stored with and without preservative and mephedrone stored unpreserved. Only MDPV and dibutylone in preserved and unpreserved and *N*-ethylpentylone in preserved whole blood were stable for the entire study, while other analytes experienced instability ranged from ~ 78% remaining (for methylone stored without preservative) to ~ 6% remaining (4-Cl-α-PVP stored with NaF).

Conversely, most metabolites were stable when stored at -20 °C until the end of the study period. 4-F-PHP and 4-MPD dihydro-metabolites, however, exhibited significant degradation (< 80% remaining) after 90 and 180 days, respectively, but with more than 60% in their contents regardless preservation.

#### Stability at − 40 °C

**Fig. 6:**
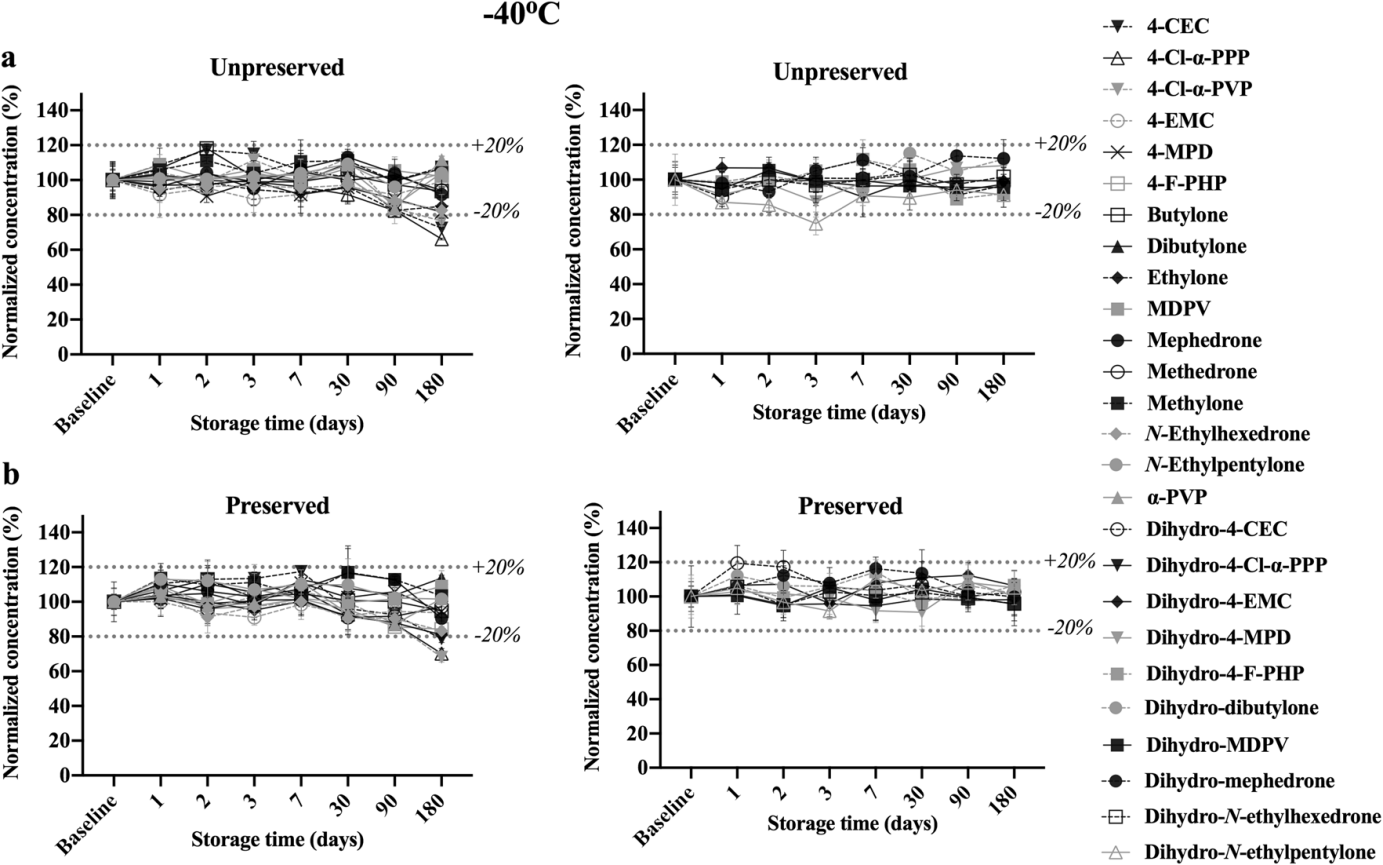
Concentrations remaining after storage at − 40 °C for 26 analytes and dihydro-metabolites at 500 ng/mL over 6 months. All concentrations were calculated as percentages of the baseline concentrations on *T*_0_. **a** Parent analytes (left) and dihydro-metabolites (right) in unpreserved whole blood. **b** Parent analytes (left) and dihydro-metabolites (right) in preserved whole blood

An illustration of stability of the analytes and dihydro-metabolites over the course of 6 months when stored at − 40 °C is given in Fig. 6. No instability was appreciable for all analytes up to 90 days of storage in both preserved and unpreserved samples. Degradation did become significant for 4-MPD, 4-Cl-α-PPP, 4-CEC, and 4-Cl-α-PVP, maintaining more than 65% of its contents, under both storage conditions. No instability was noted for any dihydro-metabolites during the whole observation period whether preserved or unpreserved.

## Discussion

The stability study described here expanded on previous studies of SCt, where only parent analytes were often examined [8], but presented new data in relation to analytically important dihydro-metabolites. It additionally examined the effects of various storage temperatures with and without the addition of the preservative NaF to assess the best storage conditions for maintaining SCt stability in whole blood samples. Pooled whole blood was utilized to assure that any effect from independent donors would not impact on the stability results, although it is possible that stability may vary depends on individual donor samples.

The best stability was observed with all SCt parent analytes and dihydro-metabolites when stored frozen, which was expected based on other SCt stability studies and what is well known about the influence of temperatures on analytes’ stability. Among frozen conditions, all 26 parent analytes and dihydro-metabolites in whole blood samples showed better stability at − 40 °C than at − 20 °C. In comparing frozen conditions at − 20 and − 40 °C, there was a statistically significant difference in stability (*p* < 0.05), regardless of preservation. Significant degradation occurred for several analytes after 30 days at − 20 °C (4-CEC, 4-EMC and 4-F-PHP, α-PVP, *N*-ethylhexedrone, methedrone, 4-Cl-α-PVP), 90 days (4-MPD) and 180 days (methylone and butylone), while these analytes remained stable at − 40 °C for the entire study. In line with the trend observed for the parent analytes, all dihydro-metabolites demonstrated greater stability at − 40 °C, whether preservative was present or not, as concentrations remained > 80% for the whole study period. However, storage at − 20 °C displayed instability for fewer dihydro-metabolites (e.g., 4-F-PHP and 4-MPD dihydro-metabolites), with a difference in stability between the two frozen conditions being not statistically significant (*p* > 0.05). In contrary to what was noticed in frozen samples, SCt have been found to be extremely unstable in the whole blood when stored at RT, followed by refrigeration. This pattern was observed for all analytes and dihydro-metabolites included in this study, but also varied based on their chemical structure features.

As reported in previous studies [8, 16], this work demonstrated that the stability of these analytes was highly influenced by their chemical structures, and greater stability is generally observed in the order for compounds endowed with an *N*-pyrrolidine and methylenedioxy groups (e.g., MDPV), followed by SCt substituted by *N*-pyrrolidine (e.g., α-PVP), secondary amines with methylenedioxy group (e.g., butylone, dibutylone, ethylone, methylone), and finally secondary amines with aliphatic ring substitution (e.g., mephedrone, 4-EMC, and *N*-ethylhexedrone). However, the present study has shown that the presence of an aromatic halogen translates to poor stability regardless of their group, which is a possible explanation for the difference in observed stability between 4-Cl-α-PVP and α-PVP. Our study agreed with those reported by Antunes et al*.* [17], who found that 4-CEC and 4-Cl-α-PVP to be highly unstable in whole blood samples. Although both butylone and dibutylone are structurally related, butylone was not detected after 30 days of storage at RT, while dibutylone remained detectable after 6 months of storage either without or with preservative (19.6–36.7%). It is reasonable to assume that the presence of an additional *N*-methyl group could result in greater stability for dibutylone over butylone, likely as a result of increased stability over enzymatic and/or chemical oxidation processes. Similar observation was described in other stability studies where the presence of an additional methyl group provided better resistance to degradation [18].

In unpreserved whole blood [19], after 6 weeks of storage at − 20 °C, methylone, ethylone, methedrone, butylone, mephedrone, and MDPV showed good stability with more than 80% remaining intact. In agreement with a previous study [19], our data showed acceptable stability for methylone, MDPV, and butylone over 90 days of storage at − 20 °C. However, our results demonstrated instability of ethylone, mephedrone, and methedrone as early as 30 days at − 20 °C. Johnson and Botch-Jones [20] studied the effect of storage conditions on the stability of mephedrone and MDPV in unpreserved whole blood. The data showed ~ 30% to 100% loss of mephedrone at 4 °C and RT after 7 days, respectively, while it remained stable at − 20 °C. On the other hand, MDPV remained stable under all conditions. Our data are very consistent with the results for mephedrone and MDPV published by Johnson and Botch-Jones [20].

The positive influence of NaF addition to whole blood samples on the stability of SCt was noticeable at RT only *(p* < 0.05), while the rate of degradation was statistically insignificant in samples stored refrigerated and frozen. In RT and refrigerated human blood with 1.67% NaF, Busardò et al*.* [9] observed 22 and 36.4% remaining mephedrone after 31 days, respectively, while unpreserved whole blood exhibited 100% loss at RT and 28.8% remaining at 4 °C. On the other hand, in frozen whole blood with and without NaF, they observed slight changes in concentrations with 96.9% and 95.6% remaining in preserved and unpreserved whole blood, respectively. These data follow the stability trend seen for mephedrone in our study, where the influence of NaF was more pronounced at higher temperatures, although the overall stability seemed to be poorer in our study than that previously reported for preserved whole blood. In good agreement with the results of our study, Czerwinska et al*.* [10] observed 76.6–85.8% and 97.4–98% (at low and high QC, respectively) remaining mephedrone levels after 10 days in refrigerated and frozen whole blood, respectively, containing 0.3% NaF/KOx.

With the complete loss of certain parent SCt especially at RT and 4 °C, there was no sampling point and storage condition where dihydro-metabolites were undetectable. For instance, degradation of parent mephedrone started as early as 1 day of storage at RT and was not detected after only 7 days either with or without preservative, but in comparison to the corresponding reduced oxo-metabolite, dihydro-mephedrone was more stable overall. It can be assumed that the transformation into a hydroxyl group resulting from ketone functional group reduction may be responsible for the observed dihydro-metabolites stability, as other authors clarify that this group is resistant to degradation [11, 21]. Czerwinska et al*.* [10] corroborated these findings, since they reported that reduced metabolites (i.e., dihydro-mephedrone and dihydro-normephedrone) were fairly stable for 10 days at either 4 °C and − 20 °C, unlike their parent analyte.

From an analytical perspective, given the extreme instability of numerous parent cathinones, specifically those featuring halogenated groups, the detection of these instability products and metabolites in the blood might be useful biomarkers and would greatly increase the detection windows for suspected SCt use. Indeed, dihydro-metabolites continue to be detected in forensic caseworks either in the presence or absence of parent drug [22–25].

While the findings of this study present important considerations to prevent/slow potential loss of analytes concentrations, there are some limitations of the study. Pooling all the parent analytes and metabolites in one sample may hinder the ability to assess potential conversions resulting from in vitro instability (e.g., dehalogenation to the dehalogenated analogues, and/or reduction of parent SCt to their corresponding dihydro-metabolites). Moreover, the pH of the whole blood samples was not monitored throughout the study; therefore, it is unknown whether changes in pH had any effect on the stability of analytes.

## Conclusions

The present study covers an observation period of 6 months, showing that storage temperatures and the chemical nature of the SCt impact the stability of SCt to a greater extent than addition of preservative in whole blood samples. Addition of NaF (2% w/v) to whole blood samples could be useful to slow degradation, especially at RT. Although greater stability is generally observed in compounds endowed with an *N*-pyrrolidine group, in cases where aromatic halogens are suspected to be present, significant degradation should be expected. Results from dihydro-metabolites demonstrated greater stability under all conditions which can prompt their preferred use as biomarkers of SCt consumption. Samples should be analyzed as soon as possible after collection to ensure accurate interpretation of toxicological results. Data after prolonged storage suggest that samples are best stored frozen at − 40 °C (or lower).

## Supplementary Information

Below is the link to the electronic supplementary material.

Supplementary file1 (DOCX 90 KB)
