# Supplementary material for: Influence of long-term storage temperatures and sodium fluoride preservation on the stability of synthetic cathinones and dihydro-metabolites in human whole blood
Source: Forensic Toxicol. 2022 Aug 6;41(1):81–93. doi: 10.1007/s11419-022-00634-w (PMC9849191; doi:10.1007/s11419-022-00634-w)
Supplement: Supplementary file 1 — Supplementary file1 (DOCX 90 KB) [file 11419_2022_634_MOESM1_ESM.docx]

**Supplementary material**

**Influence of long-term storage temperatures and sodium fluoride preservation on the stability of synthetic cathinones and dihydro-metabolites in human whole blood**

**Abdulaziz A. Aldubayyan ^1,2^, Erika Castrignanò ^1^, Simon Elliott ^1,3^, Vincenzo Abbate* ^1^**

**^1^ Department of Analytical, Environmental & Forensic Sciences, Faculty of Life Sciences & Medicine, King’s College London, London, UK**

**^2^ Department of Toxicology, Central Military Laboratory and Blood Bank, Prince Sultan Military Medical City, Riyadh, Saudi Arabia**

**^3^ Elliott Forensic Consulting, Birmingham, UK**

***Corresponding author: Dr Vincenzo Abbate; vincenzo.abbate@kcl.ac.uk**

**Table S1** Group A and B of controls comprising of common drugs (*n* = 196) at a concentration of 10 or 100 ng/mL (depending on drug)

| Analytes in Group A | | | | | | |
| --- | --- | --- | --- | --- | --- | --- |
| - Acebutolol - Alprenolol - Atenolol - Betaxolol - Bisoprolol - Bopindolol - Bunolol - Carteolol - Carvedilol - Celiprolol - Esmolol - Labetalol - Metipranolol - Metoprolol - Nadoxolol - Oxprenolol - Pindolol - Propranolol - Sotalol - Timolol - Stanozolol - Danazol - Gestrinone - Metribolone - Tetrohydrogestrinone - Clenbuterol | | - Tibolone - Zilpaterol - Bambuterol - Fenoterol - Formoterol - Salbutamol - Terbutaline - Aminoglutethimide - Anastrazole - Testolactone - Tamoxifen - Toremifene - Amiloride - Bumetanide - Canrenone - Chlorexolone - Clopamide - Indapamide - Triamterene - Amfepramone - Amiphenazole - Amphetamine - Benzoylecgonine - Benzphetamine - Benzylpiperazine - *p*-OH amphetamine | | - Cathine - Ephedrine - Pholedrine - Cropropamine - Crotethamide - Etamivan - Etilefrine - Fencamfamine - Fencamine - Fenetylline - Fenfluramine - Fenproporex - Heptaminol - Hydroxybromant. - Isometheptene - Methylenedioxyamphetamine - Ecstasy - Mefenorex - Mesocrab - Methamphetamine - Ortetamine - Phentermine - Methoxyphenamine - Methylephedrine - Methylhexaneamine | | - Modafinil - Nikethamide - Norfenfluramine - Pemoline - Cyclazodone - Famprofazone - Pentetrazol - Phendimetrazine - Prolintane - Ritalinic acid - Selegiline - Sibutramine - Strychnine - Dimethylamphetamine - Mephentermine - Dobutamine - Buprenorphine - Dextromoramide - Fentanyl - Hydromorphone - Hydrocodone - Morphine - Codeine - Methadone - Oxycodone - Pentazocine |
| *Continued* | | | | | | |
| - Oxymorphone - Pethidine - Beclomethasone - Betamethasone - Budesonide - OH-prednisolone - 6b-OH budesonide - Clobetasol - Prednisolone | | - Prednisone - Methylprednisolone - Deflazacort - Desacetyl deflazacort - Desonide - Fludrocortisone - Flumethasone - Triamcinolone acetonide - Fluticasone proprionate | | - FPCAM - Triamcinolone - Bupropion - Tramadol - Ketoconazole - Andarine - Ostarine - Acetazolamide - Chlorothiazide | | - Chlorthalidone - Dichlorphenamide - Etacrynic acid - Furosemide - Methylphenidate |
| Analytes in Group B | | | | | | |
| - Efaproxiral - Acetylcarnitine - Diacetolol - Nadolol - OH-propranolol - Exemestane - Sameterol - Clomiphene - Fulvestrant - Mefruside - Eplerenone - Piretanide - Amphetaminil - Benfluorex - Carphedon - Clobezorex - Oxilofrine | - Fenbutrazate - Phenpromethamine - *p*-Methylamphetamine - Tuaminoheptane - Adrafinil frag - Propylhexedrine - Etilamphetamine - Ethylphenylbutylamine - Prenylamine - Mephedrone - Trimetazidine - 1,3-Dimethoxybenzene - 2-Amino-6-methylheptane - 6-Monoacetylmorphine - Dexamethasone - Flunisolide - Fluocortolone | | - Pipradrol - Mitragynine - OH-mitragynine - Fluconazole - Miconazole - Itraconazole - *N,N*-Dimethyltryptamine - Selective androgen receptor modular- 4F - Letrazole metab. - Selective androgen receptor modular 4-Cl - Probenecid - Bendroflumethazide - Metolazone - Benzthiazide - Cyclopenthiazide | | - Cyclothiazide - Epitizide - Polythiazide - Trichloromethiazide - Altizide - Butizide - Methyclothiazide - Xipamide - Torasemide - Hydroflumethiazide | |

**Table S2** Calibration parameters, limit of detection, and limit of quantification for all analytes in whole blood samples

| **Analyte** | **LOD**  **(ng/mL)** | **LOQ**  **(ng/mL)** | **Linearity**  **(ng/mL)** | **Intercept ± SD (*n*=5)** | **Slope**  **± SD (*n*=5)** | ***R*^2^**  **± SD (*n*=5)** |
| --- | --- | --- | --- | --- | --- | --- |
|  |  |  |  |  |  |  |
|  |  |  |  |  |  |  |
| Mephedrone | 0.15 | 1 | 1–1000 | -0.0015 ± 0.0050 | 0.1101 ± 0.0096 | 0.9982 ± 0.0014 |
| Methylone | 0.21 | 1 | 1–1000 | -0.0126 ± 0.0071 | 0.1102 ± 0.0117 | 0.9984 ± 0.0011 |
| Methedrone | 1.45 | 5 | 5–1000 | -0.0263 ± 0.0260 | 0.0591 ± 0.0050 | 0.9987 ± 0.0008 |
| Ethylone | 0.10 | 1 | 1–1000 | -0.0097 ± 0.0039 | 0.1244 ± 0.0268 | 0.9978 ± 0.0013 |
| Butylone | 0.58 | 1 | 1–1000 | -0.0073 ± 0.0048 | 0.0272 ± 0.0050 | 0.9957 ± 0.0032 |
| Dibutylone | 0.21 | 1 | 1–1000 | -0.0003 ± 0.0107 | 0.1645 ± 0.0206 | 0.9960 ± 0.0022 |
| 4-CEC | 0.35 | 1 | 1–1000 | -0.0038 ± 0.0058 | 0.0544 ± 0.0033 | 0.9965 ± 0.0016 |
| 4-Cl-α-PPP | 1.08 | 5 | 5–1000 | -0.0501 ± 0.0595 | 0.1821 ± 0.0189 | 0.9972 ± 0.0013 |
| *N*-Ethylpentylone | 0.81 | 5 | 5–1000 | 0.0050 ± 0.0214 | 0.0868 ± 0.0063 | 0.9955 ± 0.0039 |
| 4-EMC | 0.69 | 5 | 5–1000 | -0.0056 ± 0.0184 | 0.0877 ± 0.0061 | 0.9966 ± 0.0014 |
| α -PVP | 0.14 | 1 | 1–1000 | -0.0399 ± 0.0092 | 0.2190 ± 0.0222 | 0.9968 ± 0.0028 |
| MDPV | 1.09 | 5 | 5–1000 | -0.0880 ± 0.0457 | 0.1389 ± 0.0132 | 0.9966 ± 0.0021 |
| 4-MPD | 0.10 | 1 | 1–1000 | -0.0489 ± 0.0122 | 0.4175 ± 0.0635 | 0.9970 ± 0.0022 |
| *N*-Ethylhexedrone | 0.24 | 1 | 1–1000 | -0.0191 ± 0.0129 | 0.1760 ± 0.0176 | 0.9982 ± 0.0014 |
| 4-F-PHP | 0.10 | 1 | 1–1000 | -0.0447 ± 0.0085 | 0.2770 ± 0.0321 | 0.9976 ± 0.0015 |
| 4-Cl-α-PVP | 0.21 | 1 | 1–1000 | -0.0037 ± 0.0085 | 0.1344 ± 0.0237 | 0.9965 ± 0.0017 |
| Dihydro-mephedrone | 0.27 | 1 | 1–1000 | 0.0045 ± 0.0107 | 0.1336 ± 0.0147 | 0.9958 ± 0.0010 |
| Dihydro-MDPV | 0.48 | 1 | 1–1000 | 0.0050 ± 0.0078 | 0.0541 ± 0.0104 | 0.9975 ± 0.0018 |
| Dihydro-4-Cl-α-PPP | 0.32 | 1 | 1–1000 | 0.0112 ± 0.0094 | 0.0967 ± 0.0155 | 0.9966 ± 0.0023 |
| Dihydro-4-EMC | 0.16 | 1 | 1–1000 | 0.0141 ± 0.0069 | 0.1438 ± 0.0138 | 0.9976 ± 0.0008 |
| Dihydro-*N*-ethylhexedrone | 0.17 | 1 | 1–1000 | -0.0002 ± 0.0120 | 0.2295 ± 0.0212 | 0.9979 ± 0.0024 |
| Dihydro-dibutylone | 0.24 | 1 | 1–1000 | 0.1058 ± 0.0698 | 0.9576 ± 0.0997 | 0.9970 ± 0.0023 |
| Dihydro-*N*-ethylpentylone | 0.25 | 1 | 1–1000 | 0.0126 ± 0.0103 | 0.1385 ± 0.0155 | 0.9962 ± 0.0006 |
| Dihydro-4-MPD | 0.20 | 1 | 1–1000 | 0.0676 ± 0.0420 | 0.6820 ± 0.0503 | 0.9976 ± 0.0021 |
| Dihydro-4-CEC | 0.22 | 1 | 1–1000 | 0.0048 ± 0.0049 | 0.0731 ± 0.0088 | 0.9971 ± 0.0011 |
| Dihydro-4-F-PHP | 0.11 | 1 | 1–1000 | -0.0499 ± 0.0120 | 0.3606 ± 0.0256 | 0.9962 ± 0.0021 |

*LOD* limit of detection, *LOQ* limit of quantification, *SD* standard deviation

**Table S3** Bias and precision for blood at QC low (30 ng/mL), QC medium (400 ng/mL) and QC high (800 ng/mL) concentrations

| Analyte | Nominal concentration  (ng/mL) |  | Average (*n* = 3), CV (%), bias (%) | | | | | | |  |  | Precision | Precision |
| --- | --- | --- | --- | --- | --- | --- | --- | --- | --- | --- | --- | --- | --- |
|  |  |  | Run 1  (*n* = 3) | Run 2  (*n* = 3) | Run 3  (*n* = 3) | Run 4  (*n* = 3) | | Run 5  (*n* = 3) | | Grand ave.  (*n* = 15) | Bias (%)  (*n* = 15) | CV (%) intraday  (*n* = 15) | CV (%) interday  (*n* = 15) |
| Mephedrone | 30 | CV (%)  Bias (%) | 3.6  -14.9 | 4.9  -8.8 | 0.5  -4.0 | 8.8  -5.1 | | 10.4  12.3 | | 28.8 | -4.1 | 11.5 | 12.0 |
|  | 400 | CV (%)  Bias (%) | 5.7  17.5 | 4.0  7.9 | 4.0  6.6 | 1.7  19.7 | | 7.5  14.3 | | 453 | 13.2 | 6.3 | 6.5 |
|  | 800 | CV (%)  Bias (%) | 1.5  11.2 | 5.8  6.3 | 7.7  3.3 | 1.6  7.8 | | 3.9  4.9 | | 854 | 6.7 | 4.7 | 4.7 |
| Methylone | 30 | CV (%)  Bias (%) | 1.4  -14.7 | 6.4  -4.6 | 2.7  -14.9 | 1.1  -16.6 | | 16.7  -11.5 | | 26.3 | -12.5 | 8.6 | 8.7 |
|  | 400 | CV (%)  Bias (%) | 0.9  9.6 | 4.2  8.0 | 1.5  10.5 | 5.3  10.3 | | 5.7  5.8 | | 435 | 8.8 | 3.8 | 3.7 |
|  | 800 | CV (%)  Bias (%) | 4.9  9.5 | 4.1  5.6 | 3.1  7.5 | 1.5  10.8 | | 6.5  8.2 | | 867 | 8.3 | 4.0 | 4.0 |
| Methedrone | 30 | CV (%)  Bias (%) | 0.7  -13.0 | 8.9  3.6 | 8.4  0.2 | 10.4  -1.8 | | 1.9  -12.7 | | 28.6 | -4.7 | 9.8 | 10.1 |
|  | 400 | CV (%)  Bias (%) | 4.8  11.5 | 4.4  1.2 | 3.2  15.6 | 5.5  9.5 | | 2.9  15.4 | | 443 | 10.7 | 6.1 | 6.3 |
|  | 800 | CV (%)  Bias (%) | 3.8  5.3 | 1.5  2.5 | 1.5  2.5 | 0.5  9.2 | | 8.4  -6.5 | | 823 | 2.8 | 6.5 | 6.8 |
| Ethylone | 30 | CV (%)  Bias (%) | 1.5  -5.3 | 2.4  -5.4 | 9.2  -12.1 | 8.0  -9.2 | | 12.1  -1.5 | | 28.0 | -6.7 | 7.7 | 7.7 |
|  | 400 | CV (%)  Bias (%) | 6.9  15.3 | 2.4  11.4 | 2.2  14.3 | 3.4  9.4 | | 3.9  17.8 | | 455 | 13.6 | 4.4 | 4.5 |
|  | 800 | CV (%)  Bias (%) | 1.7  8.4 | 3.4  8.5 | 2.6  6.7 | 6.8  -2.6 | | 1.6  8.3 | | 847 | 5.9 | 5.2 | 5.4 |
| Butylone | 30 | CV (%)  Bias (%) | 1.6  -15.4 | 5.4  -11.4 | 10.0  -12.1 | 0.9  -16.9 | | 6.2  -16.4 | | 25.7 | -14.4 | 6.2 | 6.1 |
|  | 400 | CV (%)  Bias (%) | 4.2  4.3 | 7.5  -9.4 | 3.9  8.9 | 6.5  8.7 | | 8.8  13.8 | | 421 | 5.3 | 9.5 | 9.9 |
|  | 800 | CV (%)  Bias (%) | 4.4  3.6 | 5.8  -2.6 | 5.4  4.6 | 2.2  5.4 | | 11.0  -4.0 | | 811 | 1.4 | 6.6 | 6.7 |
| Dibutylone | 30 | CV (%)  Bias (%) | 3.5  -10.5 | 2.2  2.7 | 0.3  2.9 | 10.1  -7.9 | | 3.3  12.0 | | 30.0 | -0.2 | 9.4 | 9.9 |
|  | 400 | CV (%)  Bias (%) | 1.6  11.6 | 6.7  12.4 | 1.0  9.0 | 8.2  9.3 | | 1.1  10.7 | | 442 | 10.6 | 4.3 | 4.2 |
|  | 800 | CV (%)  Bias (%) | 4.4  3.6 | 4.6  1.1 | 2.6  6.2 | 7.4  -7.0 | | 9.6  1.7 | | 809 | 1.1 | 6.9 | 7.0 |
| 4-CEC | 30 | CV (%)  Bias (%) | 4.9  -15.2 | 1.0  -14.2 | 6.1  -5.3 | 6.7  -14.3 | | 4.9  -9.1 | | 26.5 | -11.6 | 6.3 | 6.4 |
|  | 400 | CV (%)  Bias (%) | 5.5  12.5 | 6.3  8.9 | 10.8  15.4 | 10.8  15.4 | | 10.9  7.9 | | 448 | 12.0 | 7.3 | 7.2 |
|  | 800 | CV (%)  Bias (%) | 2.1  8.5 | 3.2  5.9 | 7.0  5.6 | 7.0  1.4 | | 9.1  -3.6 | | 829 | 3.6 | 6.4 | 6.5 |
| 4-Cl-α-PPP | 30 | CV (%)  Bias (%) | 0.7  -8.4 | 4.3  7.1 | 7.2  -5.1 | 2.6  -6.0 | | 14.4  -11.6 | | 28.3 | -5.8 | 12.2 | 12.5 |
|  | 400 | CV (%)  Bias (%) | 2.4  18.8 | 5.9  13.6 | 2.8  14.4 | 2.4  15.9 | | 1.1  9.6 | | 458 | 14.5 | 3.9 | 4.0 |
|  | 800 | CV (%)  Bias (%) | 2.5  8.8 | 4.3  6.7 | 1.1  6.1 | 3.0  6.6 | | 5.8  -6.4 | | 835 | 4.4 | 6.2 | 6.5 |
| *N*-Ethylpentylone | 30 | CV (%)  Bias (%) | 3.1  -11.5 | 1.4  0.6 | 5.4  -4.4 | 4.4  -6.6 | | 9.8  -6.5 | | 28.3 | -5.7 | 6.4 | 6.5 |
|  | 400 | CV (%)  Bias (%) | 4.2  11.5 | 0.6  14.2 | 0.8  13.6 | 4.6  13.0 | | 5.1  -0.8 | | 441 | 10.3 | 6.1 | 6.4 |
|  | 800 | CV (%)  Bias (%) | 0.9  5.4 | 2.6  8.7 | 2.4  5.3 | 1.8  2.1 | | 8.3  12.9 | | 855 | 6.9 | 5.1 | 5.2 |
| 4-EMC | 30 | CV (%)  Bias (%) | 8.2  -8.6 | 2.9  4.7 | 10.3  -3.0 | 2.3  -8.6 | | 11.3  -15.1 | | 28.2 | -6.1 | 9.8 | 10.1 |
|  | 400 | CV (%)  Bias (%) | 1.8  13.5 | 2.3  16.4 | 3.5  11.3 | 1.6  8.0 | | 4.3  13.1 | | 450 | 12.5 | 3.5 | 3.6 |
|  | 800 | CV (%)  Bias (%) | 1.3  10.6 | 3.4  3.1 | 2.3  4.5 | 0.7  -5.5 | | 9.1  11.7 | | 839 | 4.9 | 7.3 | 7.6 |
| α-PVP | 30 | CV (%)  Bias (%) | 3.4  -13.9 | 5.6  -17.6 | 7.3  -16.9 | 3.5  -17.1 | | 7.3  -0.4 | | 26.0 | -13.2 | 9.2 | 9.7 |
|  | 400 | CV (%)  Bias (%) | 6.3  6.3 | 5.1  10.4 | 3.4  9.7 | 2.4  6.0 | | 2.7  -5.8 | | 421 | 5.3 | 6.8 | 7.1 |
|  | 800 | CV (%)  Bias (%) | 2.2  5.5 | 3.9  4.7 | 4.0  6.6 | 4.0  5.0 | | 5.6  -3.8 | | 829 | 3.6 | 5.1 | 5.2 |
| MDPV | 30 | CV (%)  Bias (%) | 3.5  -11.2 | 6.2  -11.3 | 1.8  -7.6 | 8.1  -9.7 | | 9.8  -17.5 | | 26.6 | -11.5 | 6.6 | 6.7 |
|  | 400 | CV (%)  Bias (%) | 1.1  16.2 | 0.7  11.4 | 4.3  14.8 | 7.7  12.9 | | 8.6  17.6 | | 458 | 14.6 | 5.1 | 5.0 |
|  | 800 | CV (%)  Bias (%) | 4.9  1.7 | 5.4  1.8 | 5.2  4.2 | 3.9  7.1 | | 2.8  -4.3 | | 817 | 2.1 | 5.4 | 5.5 |
| 4-MPD | 30 | CV (%)  Bias (%) | 6.3  -16.8 | 5.5  -11.5 | 2.8  -12.6 | 5.6  -15.2 | | 11.2  -8.2 | | 26.1 | -12.9 | 6.9 | 6.8 |
|  | 400 | CV (%)  Bias (%) | 9.5  10.8 | 7.3  12.2 | 4.4  13.7 | 10.2  12.8 | | 9.7  -0.0 | | 440 | 9.9 | 8.6 | 8.6 |
|  | 800 | CV (%)  Bias (%) | 2.9  2.6 | 6.4  0.4 | 4.5  8.2 | 3.3  6.6 | | 6.6  -2.1 | | 825 | 3.2 | 5.7 | 5.8 |
| *N*-Ethylhexedrone | 30 | CV (%)  Bias (%) | 7.9  -12.9 | 5.9  -7.4 | 7.2  -11.2 | | 6.6  -12.5 | | 6.6  11.0 | 28.0 | -6.6 | 11.6 | 12.2 |
|  | 400 | CV (%)  Bias (%) | 4.1  11.3 | 3.2  14.6 | 3.9  13.0 | | 1.6  9.0 | | 8.3  0.8 | 439 | 9.7 | 6.0 | 6.2 |
|  | 800 | CV (%)  Bias (%) | 2.4  6.4 | 5.2  3.4 | 3.6  8.2 | | 3.1  2.0 | | 2.0  -2.8 | 828 | 3.4 | 4.8 | 5.0 |
| 4-F-PHP | 30 | CV (%)  Bias (%) | 1.6  -17.3 | 2.8  -19.0 | 2.3  -16.0 | | 2.5  -18.7 | | 8.7  -6.4 | 25.4 | -15.5 | 7.0 | 7.3 |
|  | 400 | CV (%)  Bias (%) | 1.0  8.2 | 4.2  -2.5 | 12.3  1.4 | | 12.9  -5.2 | | 2.0  13.1 | 412 | 3.0 | 9.5 | 9.8 |
|  | 800 | CV (%)  Bias (%) | 3.9  5.1 | 3.3  4.8 | 5.4  7.3 | | 2.1  3.9 | | 2.3  6.3 | 844 | 5.5 | 3.2 | 3.2 |
| 4-Cl-α-PVP | 30 | CV (%)  Bias (%) | 4.2  -16.3 | 3.1  -11.8 | 3.1  -14.5 | | 4.4  -17.0 | | 7.3  -1.1 | 26.4 | -12.1 | 8.0 | 8.4 |
|  | 400 | CV (%)  Bias (%) | 1.8  9.3 | 3.1  -2.4 | 1.0  3.1 | | 2.5  1.4 | | 4.2  4.3 | 413 | 3.2 | 4.5 | 4.7 |
|  | 800 | CV (%)  Bias (%) | 3.5  6.2 | 1.9  -10.1 | 0.4  -4.2 | | 4.5  -7.3 | | 5.7  1.8 | 778 | -2.7 | 7.1 | 7.5 |
| Dihydro-mephedrone | 30 | CV (%)  Bias (%) | 5.1  4.9 | 11.8  11.4 | 1.7  5.0 | | 4.7  11.8 | | 3.5  16.0 | 33.0 | 9.8 | 6.8 | 6.8 |
|  | 400 | CV (%)  Bias (%) | 8.8  2.1 | 1.6  14.3 | 2.9  11.1 | | 8.0  6.8 | | 12.1  3.8 | 431 | 7.6 | 7.7 | 7.7 |
|  | 800 | CV (%)  Bias (%) | 5.3  3.0 | 9.9  1.1 | 0.7  10.1 | | 1.3  7.8 | | 3.5  7.7 | 847 | 5.9 | 5.4 | 5.5 |
| Dihydro-MDPV | 30 | CV (%)  Bias (%) | 8.0  13.3 | 4.9  -17.1 | 7.4  7.5 | | 10.1  13.5 | | 12.6  -4.4 | 30.8 | 2.6 | 14.2 | 14.9 |
|  | 400 | CV (%)  Bias (%) | 4.7  10.1 | 5.1  -6.3 | 1.1  8.0 | | 5.7  14.1 | | 9.1  -12.8 | 411 | 2.6 | 11.4 | 12.1 |
|  | 800 | CV (%)  Bias (%) | 6.1  1.5 | 3.4  -5.1 | 2.8  -11.4 | | 2.3  0.7 | | 2.6  1.1 | 779 | -2.6 | 6.2 | 6.5 |
| Dihydro-4-Cl-α-PPP | 30 | CV (%)  Bias (%) | 6.6  -16.3 | 4.9  -2.3 | 6.5  -12.3 | | 0.8  -5.9 | | 4.7  -9.8 | 27.2 | -9.3 | 7.0 | 7.3 |
|  | 400 | CV (%)  Bias (%) | 6.7  4.6 | 8.4  -1.2 | 4.0  0.9 | | 3.2  9.4 | | 9.9  -2.1 | 409 | 2.3 | 7.1 | 7.2 |
|  | 800 | CV (%)  Bias (%) | 7.3  -6.4 | 1.7  -9.0 | 5.9  -9.4 | | 2.3  -1.4 | | 2.1  5.8 | 767 | -4.1 | 7.2 | 7.5 |
| Dihydro-4-EMC | 30 | CV (%)  Bias (%) | 8.9  4.3 | 3.4  6.1 | 3.0  -3.9 | | 8.1  4.3 | | 5.0  16.3 | 31.6 | 5.4 | 8.2 | 8.5 |
|  | 400 | CV (%)  Bias (%) | 4.4  16.1 | 5.9  10.7 | 6.9  17.6 | | 1.9  18.4 | | 9.7  -2.8 | 448 | 12.0 | 8.9 | 9.3 |
|  | 800 | CV (%)  Bias (%) | 2.0  4.7 | 2.2  2.4 | 0.9  8.1 | | 2.7  0.9 | | 2.5  -4.0 | 819 | 2.4 | 4.5 | 4.7 |
| Dihydro-*N*-ethylhexedrone | 30 | CV (%)  Bias (%) | 7.1  2.7 | 6.0  8.5 | 6.8  1.3 | | 1.5  -5.8 | | 10.5  11.6 | 31.1 | 3.6 | 8.6 | 8.8 |
|  | 400 | CV (%)  Bias (%) | 1.2  15.4 | 8.5  10.2 | 0.9  9.4 | | 4.1  2.9 | | 6.0  10.9 | 439 | 9.8 | 5.7 | 5.8 |
|  | 800 | CV (%)  Bias (%) | 2.6  7.0 | 2.7  1.1 | 7.7  -3.4 | | 4.8  -4.0 | | 5.0  -6.9 | 790 | -1.2 | 6.5 | 6.8 |
| Dihydro-dibutylone | 30 | CV (%)  Bias (%) | 6.4  9.6 | 2.7  16.3 | 2.3  10.7 | | 1.5  13.7 | | 6.6  3.7 | 33.2 | 10.8 | 5.4 | 5.5 |
|  | 400 | CV (%)  Bias (%) | 5.0  16.9 | 3.8  18.0 | 2.7  18.7 | | 1.6  16.4 | | 0.8  16.4 | 469 | 17.3 | 2.8 | 2.7 |
|  | 800 | CV (%)  Bias (%) | 4.0  5.6 | 1.5  7.1 | 1.9  4.8 | | 2.7  2.0 | | 5.4  1.7 | 834 | 4.2 | 3.6 | 3.6 |
| Dihydro-*N*-ethylpentylone | 30 | CV (%)  Bias (%) | 8.6  -1.2 | 7.7  -3.4 | 10.6  -7.3 | | 7.0  0.4 | | 13.1  8.6 | 29.8 | -0.6 | 9.9 | 9.9 |
|  | 400 | CV (%)  Bias (%) | 1.8  14.2 | 5.6  5.4 | 5.1  4.8 | | 2.1  13.5 | | 10.7  1.8 | 432 | 7.9 | 6.8 | 7.0 |
|  | 800 | CV (%)  Bias (%) | 6.6  2.7 | 3.2  -6.3 | 4.9  3.2 | | 3.2  -5.4 | | 4.4  5.4 | 799 | -0.1 | 6.4 | 6.6 |
| Dihydro-4-MPD | 30 | CV (%)  Bias (%) | 4.1  9.3 | 3.3  -5.9 | 8.0  13.2 | | 6.0  6.3 | | 11.5  -9.5 | 30.8 | 2.7 | 10.7 | 11.2 |
|  | 400 | CV (%)  Bias (%) | 2.0  9.6 | 2.4  4.8 | 0.8  17.3 | | 2.4  12.3 | | 5.4  13.8 | 446 | 11.6 | 4.7 | 4.9 |
|  | 800 | CV (%)  Bias (%) | 6.2  0.9 | 4.2  -0.7 | 6.3  2.2 | | 3.9  -5.3 | | 3.7  -12.0 | 776 | -3.0 | 7.0 | 7.3 |
| Dihydro-4-CEC | 30 | CV (%)  Bias (%) | 3.8  -0.1 | 4.6  8.2 | 4.4  10.4 | | 1.4  6.7 | | 9.2  5.9 | 31.9 | 6.2 | 5.6 | 5.7 |
|  | 400 | CV (%)  Bias (%) | 2.8  16.5 | 1.7  12.0 | 3.2  9.3 | | 0.9  18.3 | | 5.9  9.2 | 452 | 13.1 | 4.4 | 4.6 |
|  | 800 | CV (%)  Bias (%) | 1.2  5.1 | 1.1  3.5 | 1.7  -2.1 | | 1.5  6.7 | | 2.1  -0.8 | 820 | 2.5 | 3.7 | 3.9 |
| Dihydro-4-F-PHP | 30 | CV (%)  Bias (%) | 6.7  -14.8 | 3.8  -17.9 | 4.1  -18.3 | | 1.5  -13.7 | | 3.9  -17.1 | 25.1 | -16.3 | 4.3 | 4.2 |
|  | 400 | CV (%)  Bias (%) | 12.0  10.0 | 9.0  -3.9 | 5.5  12.2 | | 5.5  10.7 | | 2.3  6.1 | 428 | 7.0 | 8.6 | 8.7 |
|  | 800 | CV (%)  Bias (%) | 2.2  5.6 | 1.5  9.5 | 2.2  9.5 | | 3.8  3.3 | | 6.2  -2.4 | 835 | 4.4 | 4.9 | 5.1 |

*QC* quality control, *CV* coefficient of variation

**Fig. S1** Matrix effects of synthetic cathinones and metabolites in blood at 30 (displayed in blue as “low”) and 800 ng/mL (shown in red as “high”). The error bars show relative standard deviation (*n* = 5)
